# Structure of Silk I (*Bombyx mori* Silk Fibroin before Spinning) -Type II β-Turn, Not α-Helix-

**DOI:** 10.3390/molecules26123706

**Published:** 2021-06-17

**Authors:** Tetsuo Asakura

**Affiliations:** Department of Biotechnology, Tokyo University of Agriculture and Technology, 2-24-16 Nakacho, Koganei, Tokyo 184-8588, Japan; asakura@cc.tuat.ac.jp

**Keywords:** *Bombyx mori* silk fibroin, silk I, solid-state NMR, IR, β-turn

## Abstract

Recently, considerable attention has been paid to *Bombyx mori* silk fibroin by a range of scientists from polymer chemists to biomaterial researchers because it has excellent physical properties, such as strength, toughness, and biocompatibility. These appealing physical properties originate from the silk fibroin structure, and therefore, structural determinations of silk fibroin before (silk I) and after (silk II) spinning are a key to make wider applications of silk. There are discrepancies about the silk I structural model, i.e., one is type II β-turn structure determined using many solid-state and solution NMR spectroscopies together with selectively stable isotope-labeled model peptides, but another is α-helix or partially α-helix structure speculated using IR and Raman methods. In this review, firstly, the process that led to type II β-turn structure by the authors was introduced in detail. Then the problems in speculating silk I structure by IR and Raman methods were pointed out together with the problem in the assignment of the amide I band in the spectra. It has been emphasized that the conformational analyses of proteins and peptides from IR and Raman studies are not straightforward and should be very careful when the proteins contain β-turn structure using many experimental data by Vass et al. In conclusion, the author emphasized here that silk I structure should be type II β-turn, not α-helix.

## 1. Introduction

*Bombyx mori* (*B. mori)* silk fibroin continues to attract the attention of many researchers in textile technology, biochemistry, biology, polymer chemistry, biomaterials, and so on [1]. The application of silk fibroin to the biomaterial field is an especially active area because it has several excellent properties, such as high strength, high toughness, and excellent biocompatibility [2,3,4,5,6,7,8]. These excellent properties originate from the structure of silk fibroin, and therefore the structural analysis is key to the further development of silk in wider applications.

The silk fiber from *B. mori* cocoon is a twin fiber that consists of two kinds of proteins: Silk fibroin and silk sericin. The fibroin consists of a heavy (H) chain of 390 kDa and a light (L) chain of 26 kDa connected by a disulfide bond, as well as a glycoprotein (P25 (30 kDa)) [9,10,11,12], which is secreted into the posterior silk gland. The H-chain, L-chain, and P25 are thought to be assembled with the ratio of 6:6:1. The silk fibroin is then stored in the middle silk gland and coated by silk sericin. The proteins are spun out through the anterior silk gland and converted into silk fibers. To use them as biomaterial, the coated silk sericin is usually removed by degumming process [1,3,6,7,8].

**Figure 1:**
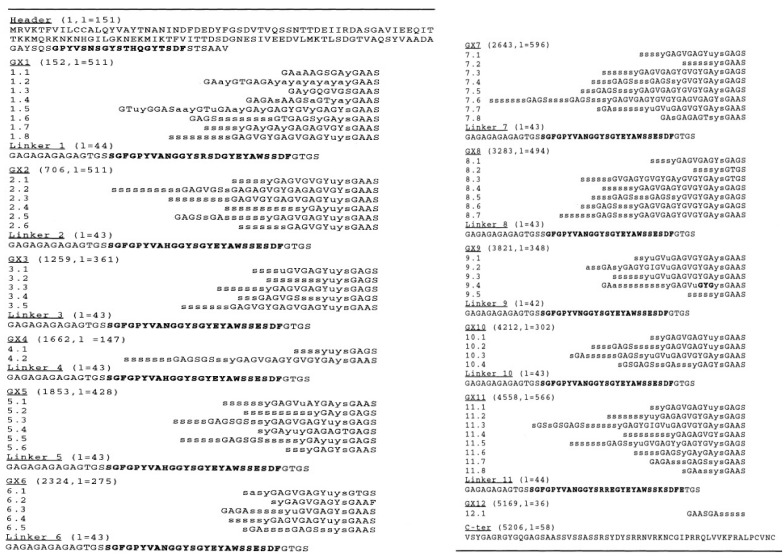
The primary structure of *B. mori* silk fibroin (H chain) was reported by Zhou et al. [13,14]. The 5263-amino acid residue chain is broken into domains and subdomains. The sequence number of the first residue and the length l of each domain are given in parentheses. The 25-residue motif in boldface characters is repeated between the header and the linkers. A one-residue (or three-residue) insertion in subdomain GX9.4 is also in boldface characters. Lower case letters s, y, a, and u represent frequently observed hexapeptides. Hexapeptides code and a number of copies; s, GAGAGS 433; y, GAGAGY 120; a, GAGAGA 27; u, GAGYGA 39. Reprinted with permission from ref. 14. Copyright 2001 John Wiley and Sons.

The H chain occupies most of the silk fibroin, and the amino acid composition is Gly (46%), Ala (30%), Ser (12%), Tyr (5.3%), Val (1.8%), etc., [13]. The primary structure of the H chain has been reported by Zhou et al. [13,14], as shown in Figure 1. The primary structure contains a large number of repeated sequences organized into 12 domains, each of which has related subdomains.

The amino acid sequence is roughly divided into four motifs: (i), (ii), (iii), and (iv) alternate along the chain [15]. Motif (i) consists of a highly repetitive AGSGAG sequence and constitutes the crystalline regions of the fiber. The total number of AGSGAG sequence repeats is 433 (2598 amino acid residues). Because the total number of amino acid residues in the H chain is 5263, almost half is the AGSGAG sequence [16]. Motif (ii) is a relatively less repetitive sequence that contains hydrophobic and/or aromatic residues GAGAGY, GAGAGV, and GAGAGVGY. Motif (iii) is very similar to the sequence of Motif (i) except for the presence of an AAS motif. Finally, Motif (iv) contains negatively charged, polar, bulky hydrophobic, and/or aromatic residues, e.g., TGSSGFGPYVANGGYSGYEYAWSSESDFGT [13,14], and makes up the amorphous regions of the silk fibroin H chain. 

It is well known that there are two kinds of crystalline forms, silk I and silk II for *B. mori* silk fibroin [17]. The former is the structure before spinning, and the latter the structure after spinning in a solid-state. Marsh et al. [18] reported that silk II was antiparallel β-sheet structure based on the X-ray diffraction analysis of silk fiber. However, most recently, the authors proposed that silk II structure was a lamella structure using ^13^C selectively labeled model peptides and solid-state NMR method [16,19]. Namely, two kinds of Ala residues in β-sheet structure, plus one distorted β-turn formed by repetitive folding using β-turns every eighth amino acid in an antipolar arrangement. However, in this review, the author will concentrate on clarifying the structure of another structure, silk I. 

The structure of silk I has been in controversy for a long time because X-ray diffraction or electron-diffraction data give only limited structural information [17,20,21,22]. More complicated situations are that the structure of silk fibroin before spinning was called as α-form at an early stage to distinguish it from β-form (silk II). Later, silk I was named instead of α-form because the name α-form may create confusion with the α-helix of Pauling and Corey [17]. However, even today, there are still many papers that the structure of silk I is α-helix or partially α-helix based on IR and Raman spectra [23,24,25,26,27,28,29,30,31,32,33,34,35,36,37]. Therefore, the author will firstly introduce the process that led to the silk I structure being type II β-turn using several many solid-state NMR and solution NMR methods together with selectively stable isotope-labeled model peptides in detail. Next, the author will emphasize the problem to conclude that the silk I structure is α-helix from IR analyses together with the assignment of the amide I band. This is based on the review by Vass et al. [38]. In conclusion, the author emphasized here that silk I structure should be type II β-turn, not α-helix.

## 2. Structure of Silk Stored in the Middle Silk Gland of *B. mori* Silkworm

**Figure 2:**
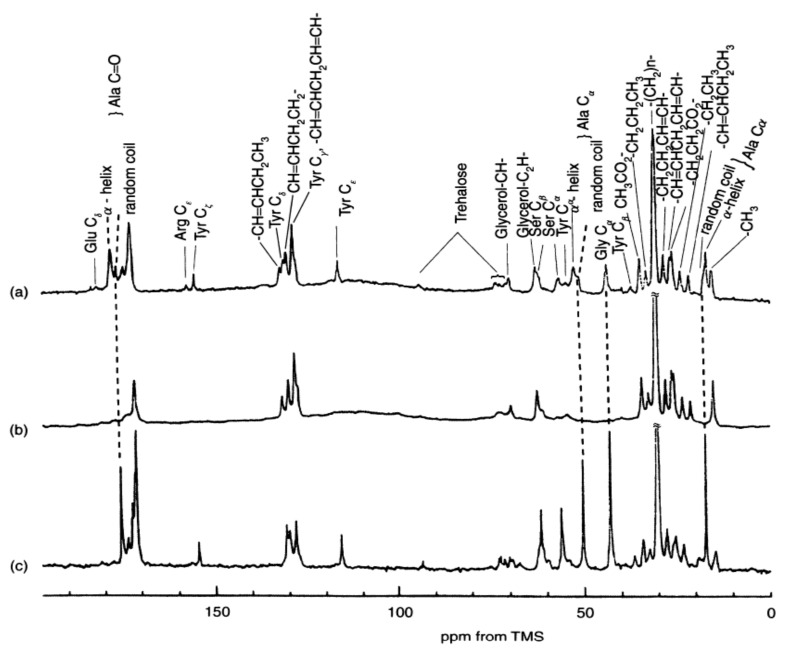
^13^C solution NMR spectra of (**a**) the middle silk gland portion of living *Samia cynthia ricini* mature larva, (**b**) the abdomen of living *S. c. ricini* pupa and (**c**) the middle silk gland portion of living *Bombyx mori* mature larva together with the assignment [39,40]. Reprinted with permission from ref. 39. Modified from ref. 39.

The authors first reported the ^13^C solution NMR spectra of liquid silks stored in the live silk moths, *Samia cynthia ricini* (*S. c. ricini*) and *B. mori* in 1983 together with the spectrum of pupa (Figure 2) [39,40]. The shapes of these 5th larval stage silk moths and pupa are suitable to put directly into the ^13^C NMR sample tube with 10 mm outer diameter. As shown in Figure 2, the ^13^C NMR peaks of the liquid silks could be obtained and easily assigned because these peaks should not be observed in the spectrum of pupa after making cocoons.

From comparing the spectra between two kinds of silkworms, it is important to emphasize that the conformation of liquid silk from *B. mori* is different from α-helix. There are mainly two kinds of Ala residues in the liquid silk of *S. c. ricini* stored in the middle silk glands, i.e., Ala residues in the polyalanine sequences (A)_n_ (n = 12, 13) and the isolated Ala residue [41]. The polyalanine sequences take α-helical conformation, whereas the isolated Ala residues take random coil in the liquid silk [39,40,42,43,44,45,46,47]. Therefore, the chemical shifts of the Ala main peaks of *S. c. ricini* liquid silk are a good chemical shift reference for α-helix, and the small peaks are a good chemical shift reference for the random coil.

As shown in Figure 2c, the chemical shifts of the single sharp peaks from Ala Cα, Cβ, and C=O carbons of *B. mori* liquid silk are quite different from the corresponding α-helical Ala peaks of *S. c. ricini* liquid silk and close to the random coil peaks. (Figure 2a) Thus, the spectral comparison of Figure 2a,c provides clear evidence against the presence of α-helix in the liquid silk from *B. mori* silkworm. The X-ray diffraction data of liquid silk dried without using any external force also clearly deny the presence of α-helix [17,18,22,48,49,50,51].

However, the in vivo NMR study does not mean that all of the *B. mori* liquid silk stored in the middle silk glands have simply random coil structure because it is expected to be hard to prepare silk fibroin fiber with high strength by silkworm all from a random coil structure. Namely, some orderly structure silk I that is not α-helix is speculated to be present in the liquid silk.

## 3. Determination of Silk I Structure in a Solid-State

**Figure 3:**
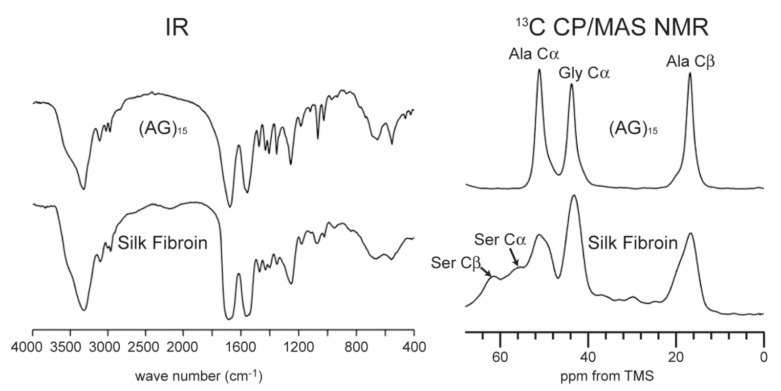
IR and ^13^C CP/MAS NMR (0–70 ppm) spectra of (AG)_15_ and *B. mori* silk fibroin with silk I structures [22]. Reprinted with permission from ref. 22.

In order to determine the silk I structure, the authors used the peptide model (AG)_15_ because the number of Ala, Gly, and Ser residues in the repeated AGSGAG sequences (crystalline region) are almost half of the total amino acid residues of silk fibroin [13,14,16]. An alternating copolypeptide (AG)_n_ has been used for the structural model of silk fibroin by X-ray diffraction analysis [17,20,48,49,50,51]. The (AG)_15_ after dialysis from 9M LiBr aqueous solution has been used as the model for silk I because the IR spectra and ^13^C chemical shifts of the main peaks from its Ala and Gly residues are the same as those from *B. mori* silk fibroin obtained by slowly drying the liquid silk taken out from silk gland at room temperature carefully, as shown in Figure 3 [21,22,52,53,54,55,56].

**Figure 4:**
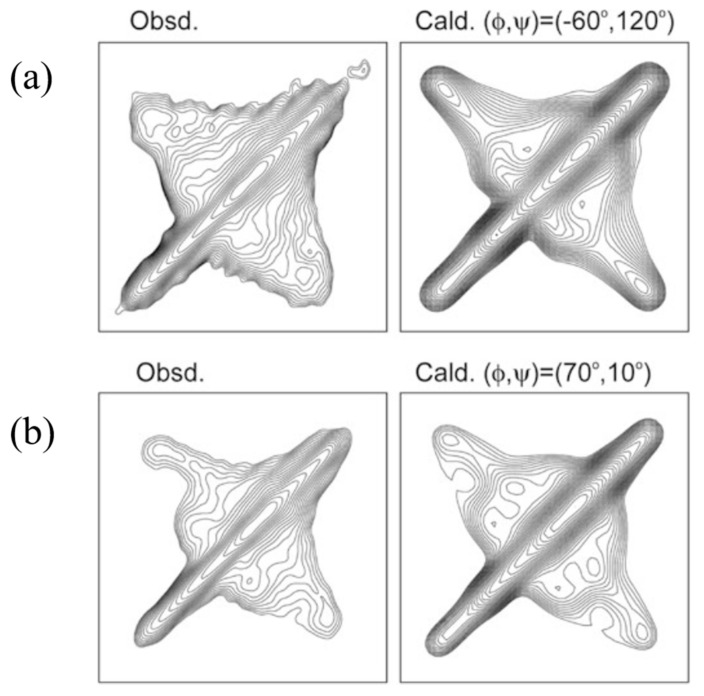
Observed 2D spin-diffusion NMR spectra of (**a**) (AG)_6_A[1-^13^C]G^14^[1-^13^C]A^15^G(AG)_7_ and (**b**) (AG)_7_[1-^13^C]A^15^[1-^13^C]G^16^(AG)_7_ with silk I structures for determination of the torsion angles (ϕ, ψ) of Ala^15^ and Gly^16^ residues, respectively. The calculated spectra are assuming the torsion angles of Ala^15^ (ϕ, ψ) = (−60°, 120°) and Gly^16^ (ϕ, ψ) = (70°, 10°) were also shown for comparison [22]. Reprinted with permission from ref. 22.

**Figure 5:**
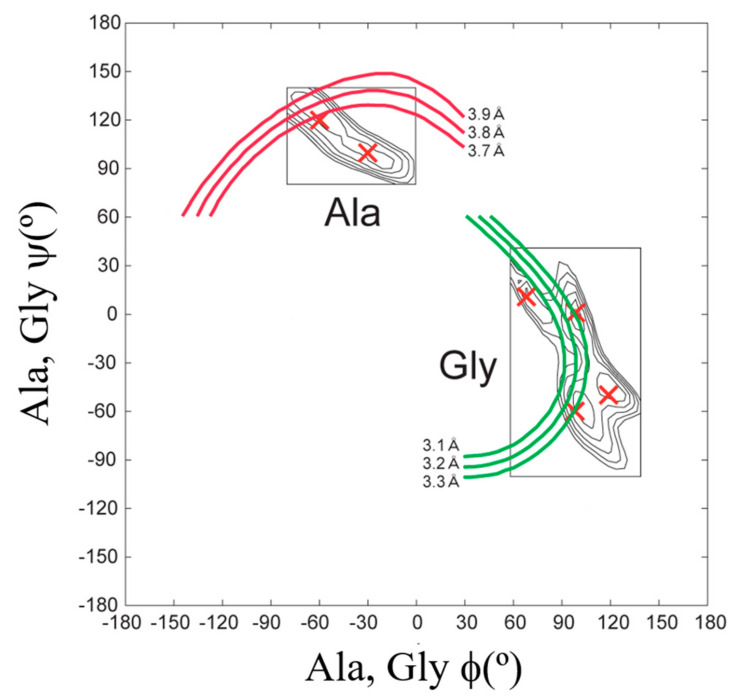
Determination of the torsion angles, (ϕ, ψ) for Ala^15^ and Gly^16^ residues in (AG)_15_ with silk I structure from both 2D spin-diffusion NMR and REDOR experiments. The candidates of the torsion angles (red X marks) were determined from the observed 2D spin-diffusion NMR spectra in Figure 4. The atomic distances between ^13^C and ^15^N nuclei were determined to be 3.8 ± 0.1 Å for [1-^13^C]G^14^A^15^[^15^N]G^16^ and 3.2 ± 0.1 Å for [1-^13^C]A^15^G^16^[^15^N]A^17^, respectively from the observed REDOR data [22]. Reprinted with permission from ref. 22. Modified from ref. 22.

To determine the atomic coordinates of the silk I structure, the authors used 2D spin-diffusion solid-state NMR and rotational-echo double-resonance, REDOR NMR spectroscopies. The 2D spin-diffusion solid-state NMR is a powerful method to obtain the relative orientation of two chemical shift tensors of ^13^C labeled sites in the local molecular framework [57,58,59,60,61]. Here, this solid-state NMR technique was used to determine the torsion angles of both silk fibroin and (AG)_15_ with silk I form [21,22,62,63,64,65,66]. The 2D spin-diffusion NMR spectra of (AG)_6_A[1-^13^C]G^14^[1-^13^C]A^15^G(AG)_7_ and (AG)_7_[1-^13^C]A^15^[1-^13^C]G^16^(AG)_7_ were observed under off magic angle spinning [21,62], and showed together with the calculated spectra in Figure 4. The errors (RMSD) between the observed and calculated 2D spin-diffusion NMR spectra were mapped in Figure 5. There are two minima, (ϕ, ψ) = (−60°, 120°) and (−30°, 100°) for Ala^15^ residue, and four minima, (ϕ, ψ) = (70°, 10°), (100°, 0°), (120°, −50°) and (100°, −60°) for Gly^16^ residue (red X marks). For further determination of the torsion angles of silk I, the REDOR method was used [21,64,65,66,67,68,69,70,71,72]. 

REDOR is an excellent method to determine the interatomic distances from dipolar couplings iny solid. The atomic distance between the C=O carbon of Gly^14^ residue and NH nitrogen of Gly^16^ residue changes significantly by changing the torsion angles (ϕ, ψ) of Ala^15^ residue, and therefore, such atomic distance information can be used for a further structural constraint. The atomic distance between [1-^13^C]Gly^14^ and [^15^N]Gly^16^ in (AG)_6_A[1-^13^C]G^14^A^15^[^15^N]G^16^(AG)_7_ could be determined as 3.8 ± 0.1 Å. Thus, the contour lines of the atomic distances, including experimental errors, are shown, and the angles (ϕ, ψ) = (−60°,120°) are selected for the Ala^15^ residue in Figure 5.

**Figure 6:**
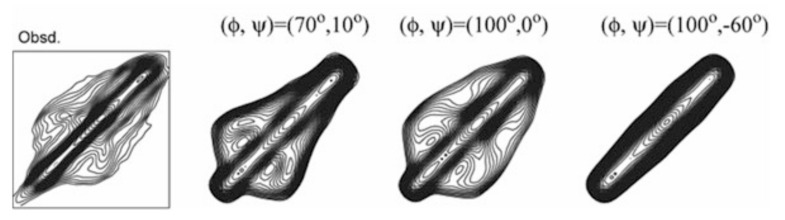
2D spin-diffusion NMR spectrum of (AG)_6_A[1-^13^C]G^14^A^15^[1-^13^C]G^16^(AG)_7_. The torsion angles of Ala^15^ residue were set as (ϕ, ψ) = (−60°, 120°), and therefore, the spectral pattern depends on the torsion angles, (ϕ, ψ) of Gly^16^. The calculated spectra for three torsion angles, (ϕ, ψ) = (70°, 10°), (100°, 0°) and (100°, −60°) selected from Figure 5 are shown [22]. Reprinted with permission from ref. 22.

A good agreement between the observed and calculated 2D spin diffusion NMR spectra was obtained when the angles, (ϕ, ψ) = (−60°, 120°), as shown in Figure 4. Next, the atomic distance between [1-^13^C]Ala^15^ and [^15^N]Ala^17^ in (AG)_7_[1-^13^C]A^15^G^16^[^15^N]A^17^G(AG)_6_ was determined as 3.2 ± 0.1 Å from the REDOR experiment. By judging from the observed atomic distance constraint, the angle (ϕ, ψ) = (120°, −50°) was ruled out in Figure 5. Then the 2D spin-diffusion NMR spectrum of (AG)_6_A[1-^13^C]G^14^A^15^[1-^13^C]G^16^(AG)_7_ was used to select one from three possible torsion angles (φ, ψ) marked by X. The spectral pattern should depend on both torsion angles of Ala^15^ and Gly^16^ residues. However, the torsion angles of Ala^15^ residue were determined as (ϕ, ψ) = (−60°, 120°) mentioned above. Therefore, the spectral pattern depends on only the torsion angle of Gly^16^ residue. Three spectral patterns were calculated for the torsion angles, (ϕ, ψ) = (70°, 10°), (ϕ, ψ) = (100°, 0°) and (ϕ, ψ) = (100°, −60°), as shown in Figure 6. By comparing the observed and calculated spectra, the torsion angles (ϕ, ψ) = (100°, −60°) could be excluded. However, it is difficult to select the torsion angles (ϕ, ψ) = (70°, 10°) or (ϕ, ψ) = (100°, 0°) because both calculated patterns can reproduce the observed spectrum well. Therefore, X-ray diffraction data of the crystalline fraction of *B. mori* silk fibroin with silk I structure reported by Lotz et al. [17,73], and Okuyama et al. [48,74] were used to determine the torsion angles of Gly^16^ residue.

The X-ray diffraction data of silk I were reported as follows. The unit cell and the space group were orthorhombic and P2_1_2_1_2_1_, respectively. The lattice constants were *a* = 4.65 Å, *b =* 14.24 Å and *c* = 8.88 Å, α = β = γ = 90°. In a unit cell, four repeats of, Ala-Gly, and two 2_1_-helix chains in an antiparallel manner are presented. Then the structure of silk I was calculated, including intermolecular chain arrangement [17,22,48,73,74,75]. The initial torsion angle for LALS (Linked-Atom Least-Squares) calculation [22,48,74] was (ϕ, ψ) = (−60°, 120°) for Ala residue, but two candidates, (ϕ, ψ) = (70°, 10°) and (ϕ, ψ) = (100°, 0°) for Gly residue as mentioned above. The calculated results are summarized in Table 1. Although the LALS calculations were performed for two cases, the same set of the torsion angles, (ϕ, ψ) = (−62°, 125°) for Ala residue and (ϕ, ψ) = (77°, 10°) for Gly residue was obtained. Namely, these torsion angles were obtained with R factor 10% independent of the initial sets of the torsion angles of Gly residues. 

**Figure 7:**
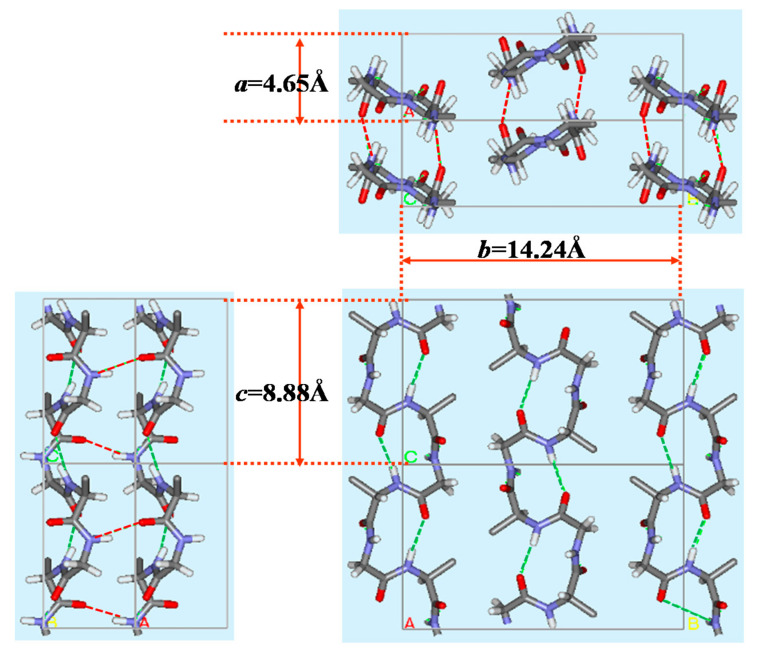
Packing structure of poly(AG) chains with type II β−turn structure as a model for silk I. Dotted lines denote intra-(green) and inter-(red) molecular hydrogen bonds. The unit lattice values, *a*, *b* and *c* were obtained from X-ray diffraction data [21,22]. Reprinted with permission from ref. 22. Modified from ref. 22.

**Figure 8:**
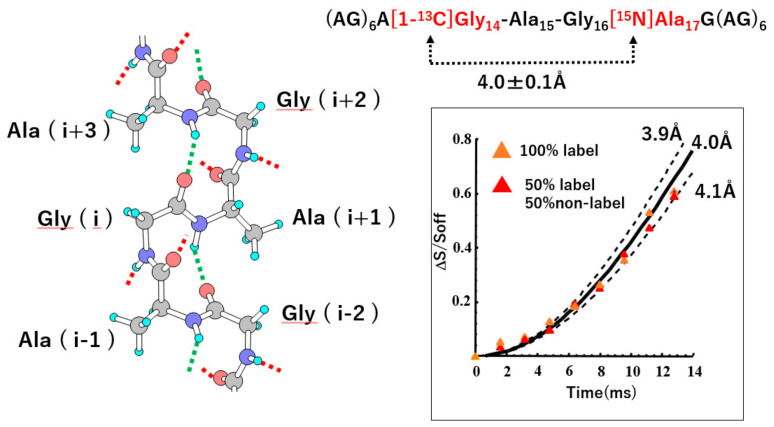
(Left) Picture of an isolated (AG)_n_ chain with type II β-turn structure. (Right) Observed plots of ΔS/S_0_ (= 1 − S/S_0_) values against the corresponding NcTr values forREDOR experiments of (AG)_6_A[1-^13^C]G^14^AG[^15^N]A^17^G(AG)_6_ to determine the distance between the [1-^13^C]Gly^14^ and [^15^N]Ala^17^ nuclei. Continuous and dotted lines show the theoretical dephasing curves corresponding to the designated distances. By comparing the REDOR data and the theoretical dephasing curve, the ^13^C-^15^N interatomic distance was determined to be 4.0 ± 0.1 Å. Reprinted with permission from ref. 21. Modified from ref. 21. Copyright 2001 Elsevier.

The final silk I structure of (AG)_n_ with intra-(green) and inter-(red) hydrogen bonds is shown in Figure 7 [21,22,66]. The most definite evidence of type II β-turn structure is the presence of an intramolecular hydrogen bond formation (green) between the carbonyl oxygen atom of the (i)-th Gly residue and the amide hydrogen atom of the (i + 3)-th Ala residue in Figure 8.

Therefore, REDOR experiment was performed to determine the atomic distance between [1-^13^C]Gly^14^ and [^15^N]Ala^17^ nuclei in (AG)_6_A[1-^13^C]G^14^A^15^G^16^[^15^N]A^17^G(AG)_6_. The observed distance was 4.0 ± 0.1 Å, independent of the dilution of natural abundance (AG)_15_, as shown in Figure 8. The corresponding distance of these atoms was calculated to be 3.9 Å in the silk I model of Figure 7, which agrees with the observed distance, 4.0 ± 0.1 Å. This short distance supports the formation of an intramolecular hydrogen bond between these two groups and type II β-turn structure clearly.

The type II β-turn structure is characterized by the alternating sequence of X-Gly, where X is another amino acid residue [76]. Namely, if the (i)-th position in the β-turn type II structure shown in Figure 8 is occupied by the residues other than Gly residue, the conformation of the backbone chain will be energetically unfavorable, due to steric hindrance between the methyl group of the (i + 3)-th Ala and the side chain of the (i)-th residue. Thus, to form the type II β-turn structure, the primary structure of the crystalline regions in *B. mori* silk fibroin should be an alternative copolymer, X-Gly. This means a close relationship between the primary and secondary structures of silk fibroin before spinning. In addition, this type II β-turn structure is easy to form lamella structure (silk II) with distorted β-turn formed by repetitive folding using β-turns every eighth amino acid in an antipolar arrangement as reported previously by authors [16,19].

## 4. Silk I Structure Determined from Solution NMR 

Solution NMR was used to determine the solution structure of *B. mori* silk fibroin before spinning. The liquid silk was obtained by removing it from the middle silk gland [39,40,63,75,76]. The uniformly ^13^C-labeled liquid silk was biosynthetically prepared by feeding U-^13^C D-glucose in addition to an artificial diet to silkworm larvae. Several 3D NMR spectra were observed to assign the NMR spectra and obtain the chemical shift data of backbone ^13^Cα, ^13^Cβ, ^13^CO, ^1^Hα, ^1^HN, and ^15^N nuclei.

**Figure 9:**
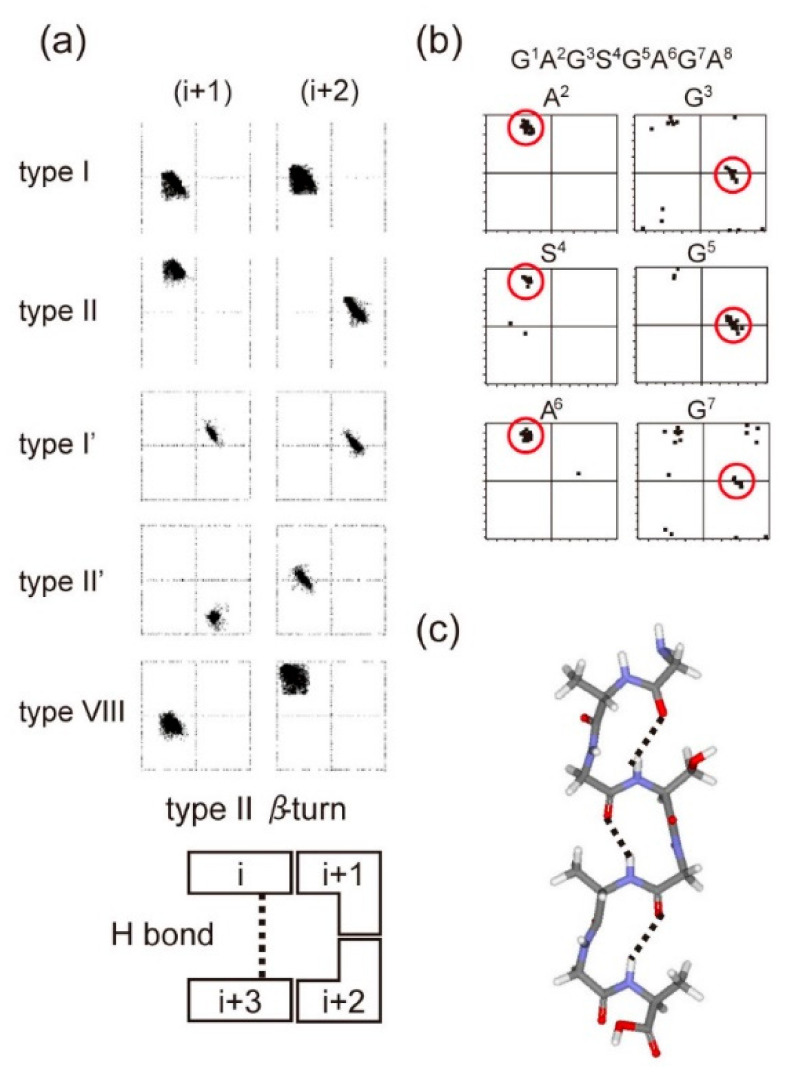
The relationship between the torsion angles (ϕ, ψ) of the (i + 1) and (i + 2)-th residues in Figure 8 and several types of β-turn structures. (**a**) The (ϕ, ψ) maps for typical type I, type II, type I′, type II′, and type VIII β-turns and an illustration of the type II β-turn conformation. (**b**) The 25 best matches for torsion angles (ϕ, ψ) of each residue of the GAGSGAGA motif obtained from the observed ^13^C solution NMR chemical shifts of *B. mori* liquid silk using the TALOS-N program [77,78]. (**c**) The structural model constructed using the averaged (ϕ, ψ) angles in the red circles in (**b**) for each motif. Hydrogen bonds are assumed to exist between the CO of the (i)-th and the NH of the (i+3)-th residues for the GAGSGAGA motif [76]. Reprinted with permission from ref. 76.

The torsion angle constraints for the main chain of silk fibroin were derived from the backbone chemical shifts using TALOS-N [77,78]. Moreover, to obtain spatial interproton distance information, the intensities of inter residue NOE cross-peaks were obtained. The combinations of torsion angle constraints determined from NMR data are consistent with only repeated type II β-turn structure among several types of β-turn structures for the tandem repeated sequences (GAGSGA)_n_ of *B. mori* silk fibroin (Figure 9) [76]. This structure determined from solution NMR is essentially the same as silk I structure in a solid-state determined above (Figure 7).

The local structure of each residue is similar to that of random coil peptides [79,80,81,82], but is more ordered. Namely, the structural fluctuations of Gly, Ala, and Ser residues are relatively large in random coil form of silk fibroin in the diluted aqueous solution, but the fluctuations decrease, due to aggregation of the silk fibroin molecules with increasing the concentration [75]. This is consistent with an increased population of silk I [83]. In other words, the highly concentrated silk solution contained in the middle silk glands has residues in energetically favored conformations close to average random coil values [39,40], but forms a hydrogen-bonded network that keeps it in a repeated type II β-turn structure [21,22]. These data and insights became the starting point to understand the molecular mechanisms behind the flow behavior generating the high-performance silk fibers from viewpoints of rheology [83,84,85].

## 5. Verification of Silk I Structure (Type II β-Turn)

At first, verification of type II β-turn structure of silk I proposed here was performed from a comparison of previous silk I models. So far, silk I structure has been studied by the combination of X-ray diffraction studies and conformational energy calculations of silk I by Lotz and Keith [17,73], Okuyama et al. [48,74], and Fossey et al. [51] without using the simple IR data. In the Lotz and Keith model [17,73], the Ala residues are in a β-sheet structure and Gly residues in a left-handed or right-handed α-helical conformation, i.e., Ala (ϕ = −105° and ψ = 112°) and Gly (ϕ = 80° and ψ = 50°) or Ala (ϕ = −125° and ψ = 88°) and Gly (ϕ = −50° and ψ = −76°). The Fossey model has a right-handed and left-handed twisting of sheets, with approximately equal magnitudes of the twist, Ala (ϕ = −80° and ψ = 150°) and Gly (ϕ = −150° and ψ = 80°). The Okuyama model is Ala (ϕ = −110° and ψ = −2°) and Gly (ϕ = 73° and ψ = −102°). Therefore, the 2D spin diffusion NMR spectra were calculated with the torsion angles of each model and compared with the observed spectra shown in Figure 4. The agreement of the calculated spectral pattern with the observed spectrum was poor for all these models [22] (Data not shown), and only the type II β-turn model proposed by authors can reproduce the observed spectrum very well (Figure 4).

Next, the structure of silk I, including the intermolecular arrangement proposed in Figure 7, was examined from the determination of accurate ^1^H positions of the structure and the theoretical chemical shift calculation of the ^1^H, ^13^C, and ^15^N nuclei [86]. Namely, the coordinates of heteroatoms, such as C, N, and O atoms, of type II β-turn structure of silk I were determined, as shown in Figure 7. However, the coordinate of H atoms was uncertain. Because H atoms are located on the surface of the silk fibroin molecule, the coordinates of H atoms are sensitive to the intra- and intermolecular arrangement. Therefore, it is important to determine the position of the H atom, but ^1^H NMR spectra in a solid-state are generally very broad contrary to ^1^H solution NMR spectra [76] because of strong dipolar coupling. The authors have developed a 1 mm micro-coil MAS NMR probe-head with high speed spinning [87]. Therefore, this micro-coil MAS NMR probe-head with a high spinning rate of 70 kHz was tried to observe a well-resolved ^1^H NMR spectrum of (AG)_15_ using an ultrahigh field NMR spectrometer at 920 MHz. 

**Figure 10:**
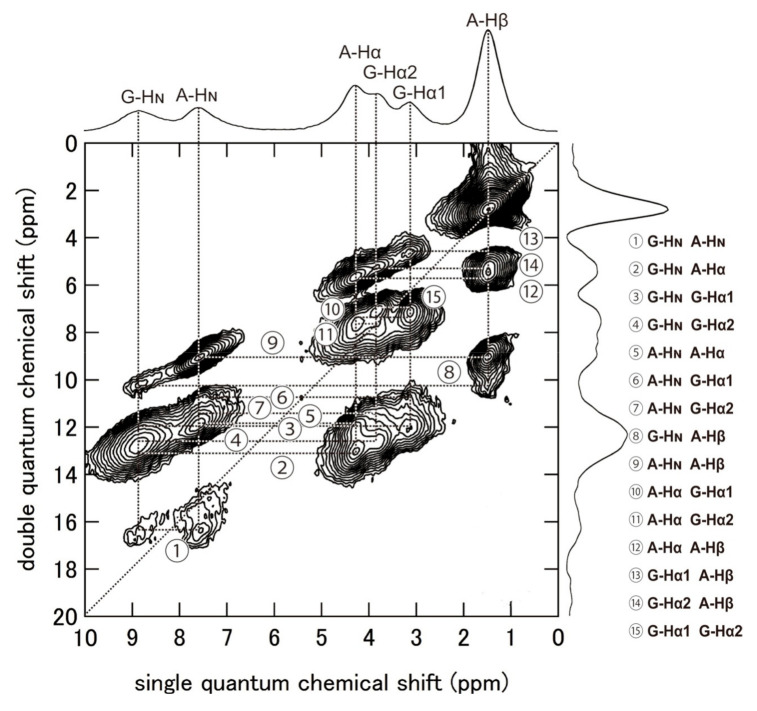
2D ^1^H (920 MHz) DQMAS NMR spectrum and the skyline projection of (AG)_15_ with silk I structure together with assignments. The DQ correlations ①–⑮ are shown on the right-side [86]. Reprinted with permission from ref. 86. Modified from ref. 86.

**Figure 11:**
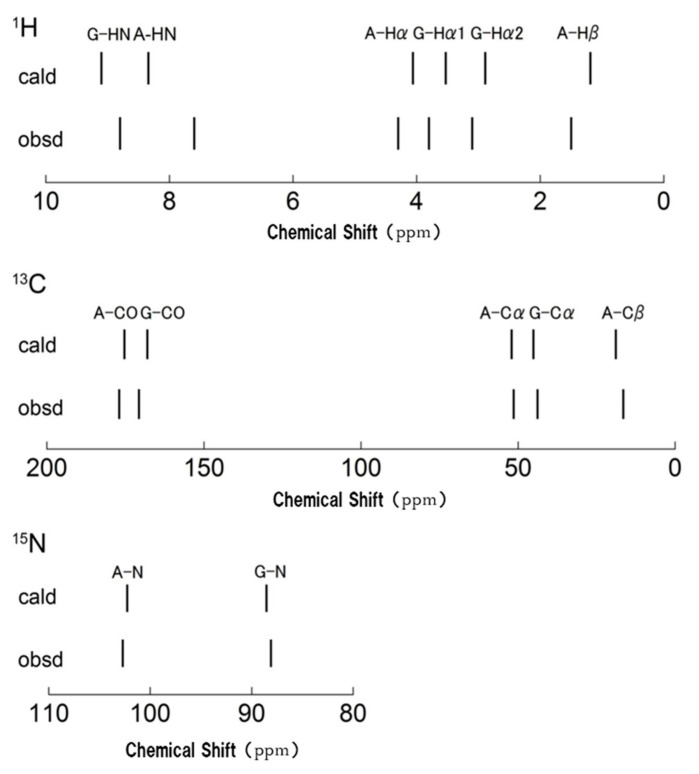
The stick spectra for the ^1^H, ^13^C, and ^15^N chemical shifts were calculated for (AG)_15_ with silk I structure together with the observed chemical shifts [86]. The reference chemical shift was shown to be the mean of the calculated and observed chemical shifts for all peaks. The values were 30.51 ppm, 171.31 ppm, and 197.22 ppm for ^1^H, ^13^C, and ^15^N nuclei, respectively. Reprinted with permission from ref. 86.

Figure 10 shows the ^1^H DQMAS (double quantum magic angle spinning) NMR spectrum of (AG)_15_ with silk I structure together with Double-Quantum (DQ) correlations to obtain information on the intra- and intermolecular ^1^H-^1^H distances in (AG)_15_ [86,88,89,90,91,92,93,94,95,96]. Thus, the ^1^H chemical shifts of (AG)_15_ with silk I structure could be obtained in a solid-state from Figure 10 with high accuracy. To calculate the coordinates of ^1^H atoms, the geometry optimization was applied only for protons under periodic boundary conditions after the atomic coordinates of the heteroatoms were fixed. All calculations were carried out by the NMR-CASTEP program. Then the ^1^H, ^13^C, and ^15^N chemical shift calculations were performed by GIPAW methods [97]. The observed of the ^1^H, ^13^C, and ^15^N chemical shifts of (AG)_15_ with silk I structure [66,81,86] were compared with those calculated by theoretical chemical shift calculation using all the coordinates of heteroatoms, such as C, N, and O atoms (Figure 7), and those of H atoms, determined here. The agreement between the calculated and observed chemical shifts of all ^1^H, ^13^C, and ^15^N nuclei of (AG)_15_ with silk I structure was excellent, as shown in Figure 11. For example, the correlation coefficient was 0.99745 for ^1^H and 0.99998 for ^13^C. Thus, the excellent agreement suggests strongly that the coordinates of H atoms of (AG)_15_ in the silk I form after CASTEP calculation could be determined with a high degree of accuracy, as well as the support of the coordinates of heteroatoms, such as C, N, and O atoms, in Figure 7. 

**Figure 12:**
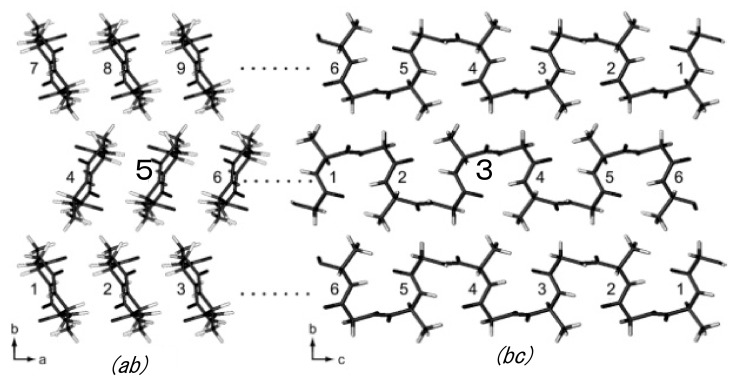
Intra- and intermolecular chain arrangements of (AG)_15_ molecules with type II β-turn structure (Figure 7) in calculating the ^1^H-^1^H distances for the DQ correlations ①–⑮ in Table 2 using coordinates of the ^1^H atoms. For example, in the column ①, G-H_N_ A-H_N_ 2.83 (5 3) in Table 2 means that the ^1^H-^1^H distance is calculated to be 2.83 Å between H_N_ Gly and H_N_ Ala residues in the chain number 5 (bold) in the group (*ab*) and the number of AG segment 3 (bold) in the group (*bc*). In addition, G-H_N_ A-H_N_ 3.43 (4 3) in Table 2 means that the ^1^H-^1^H distance is calculated to be 3.43 Å between H_N_ Gly and H_N_ Ala residues in the chain number 4 in the group (*ab*) and the number of AG segment 3 in the group (*bc*). Reprinted with permission from ref. 86. Modified from ref. 86.

Next, the intra- and intermolecular ^1^H-^1^H distances less than 4 Å were calculated from the coordinates of ^1^H atoms of the packed poly(AG) chains determined here as listed in Table 2. Figure 12 shows the intra- and intermolecular chain arrangements of (AG)_15_ molecules with type II β-turn structure in calculating the ^1^H-^1^H distances for the DQ correlations ①–⑮ in Table 2. For example, in the column ①, DQ correlation (① G-H_N_ A-H_N_) and intramolecular (2.83 (53)) means that the intramolecular ^1^H-^1^H distance between HN Gly and HN Ala residues is calculated to be 2.83 Å in the chain number 5 (bold) in the group (*ac*) and the number of Ala-Gly segment 3 (bold) in the group (*bc*). 

Although the authors did not obtain the ^1^H DQ build-up curves for quantitative simulation, the relationship between the observed DQ correlation and relative proton-proton proximities from the coordinates of (AG)_15_ with silk I form could be obtained qualitatively. A detailed analysis of the DQ correlations showed that the relative DQ peak intensities are a reliable measure of the relative distances [95,96]. The ^1^H DQMAS spectrum indicates that all the ^1^H-^1^H distances predicted in Table 2 could be observed in Figure 10. 

The geometry of the hydrogen bonding arrangements was then examined from the coordinates of ^1^H atoms of the silk I structure. The intramolecular hydrogen bonding distance was determined to be 2.01 Å between the NH of (i + 3)-th Ala residue and CO of (i)-th Gly residue. In addition, the intermolecular hydrogen bonding distance was determined to be 1.85 Å between the NH of (i + 2)-th Gly residue in one chain and CO of (i + 1)-th Ala residue in another chain. On the other hand, the angles between NH and CO bonds that contribute to the direct hydrogen bonding formation were determined as 123.2° and 168.1°, respectively. Especially, the angle, 123.2° is far from the preferred linearity for a hydrogen bond. Thus, it is easily speculated that the intermolecular hydrogen bonding is remarkably stronger than the intramolecular hydrogen bonding from the structural information about both the distance and angle of hydrogen bonding. The plane of the NH-CO peptide, and the intra- and intermolecular hydrogen bonding appear alternatively and is perpendicular to each other. Thus, it is possible to speculate that the intramolecular hydrogen bonding of the silk I structure is destroyed easily by external forces, such as stretching.

## 6. Problems in Speculating Silk I Structure from the IR Spectrum

IR is the most commonly used method to characterize the secondary structures of SF because it is installed in most research laboratories and can be measured easily [37]. In addition, the IR spectrum is possible to analyze using automated analysis carried out with commercial software (for example, Opus 6.5 software, Bruker Optics Corp., Billerica, MA, USA). Of course, it is important to use IR spectroscopy to characterize the structure of silk after the preparation of silk samples or modified silk samples with several forms for using them in many areas of use. However, the conformational analyses of proteins and peptides from IR and Raman studies are not straightforward and should be very careful when the proteins contain β-turn structure, as pointed out by Vass et al. [38]. Namely, they collected many IR experimental data about the amide I band of small and midsize peptides, cyclic peptide, and proteins with β-turn structures. Here, the amide I band carbonyl stretching coupled with in-plane NH bending and CN stretching modes) is the most intense band in the IR spectrum of silk and has been used most frequently in the conformational analysis of silk fibroin, including silk I [23,24,25,26,27,28,29,30,31,32,33,34,35,36,37]. The conclusion by Vass et al. is as follows. In the case of proteins without β-turns, IR is a reliable method for assessing secondary structures, especially with a relatively high β-sheet content. However, the prediction of β-turn content in proteins from IR spectra is not straightforward. The assignment of the 1690–1660 cm^−1^ band to β-turn in the case of proteins is still valid, provided that the contribution of β-sheet and 3_10_-helix structures are taken into account. Fortunately, the intensity of the 1690–1670 cm^−1^ component band associated with β-sheet content is week, and the content of 3_10_-helix in proteins is usually negligible. Therefore, an intense band in the 1690–1660 cm^−1^ region is highly diagnostic of turn structures. Although a string band may indicate β-turn structures, it does not always lead to accurate secondary structure estimates. Recent results on proteins indicate that β-turns also absorb in the 1645–1635 cm^−1^ region. Whether the presence of both the 1690–1660 and 1645–1635 cm^−1^ bands are required for the identification of β-turns in proteins remains to be investigated further.

**Figure 13:**
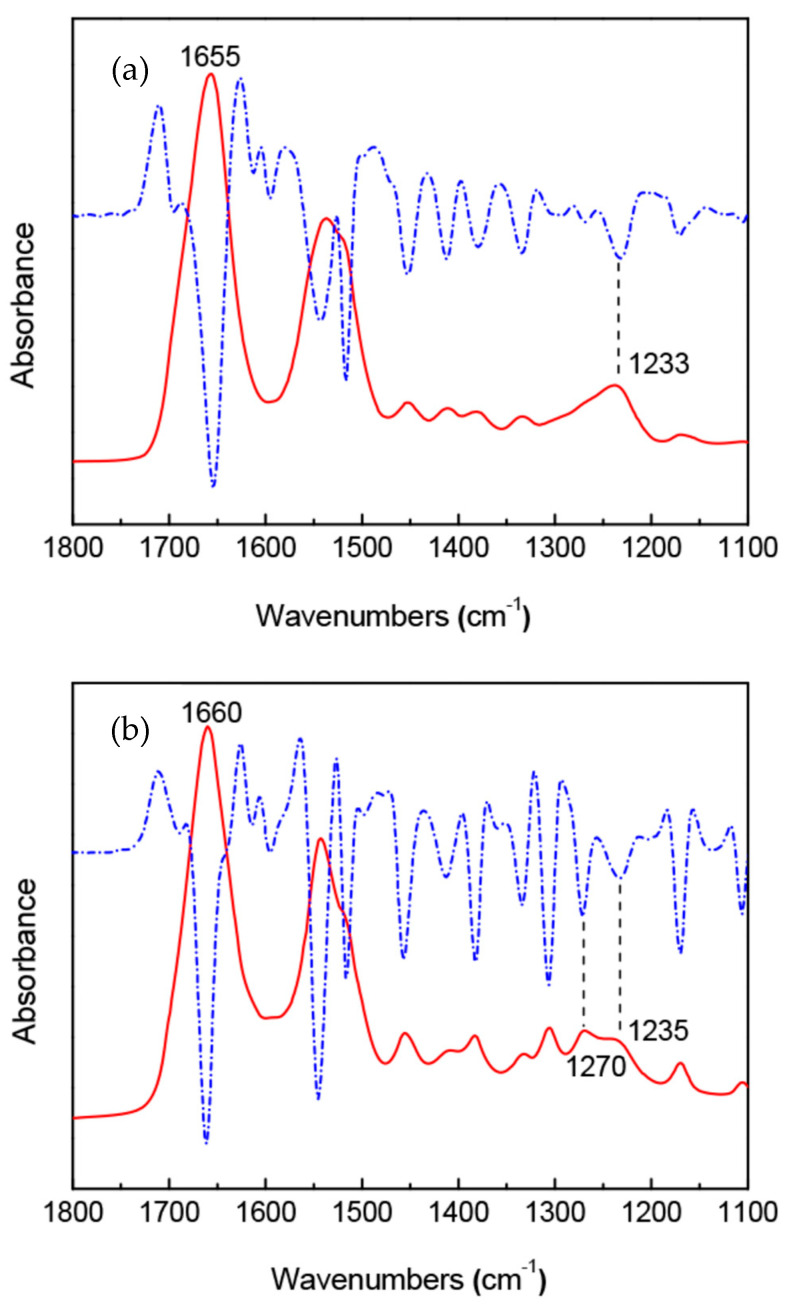
FTIR (solid line) and second derivative (dashed line) spectra of (**a**) *B. mori* silk fibroin membrane as cast with silk I structure and (**b**) *A. pernyi* silk fibroin membrane as cast [36,37]. Reprinted with permission from ref. 36.

Figure 13 shows the IR spectra of *B. mori* silk fibroin membrane as cast (silk I form) and *A. pernyi* silk fibroin membrane as a cast [36,37]. Here, the IR spectrum of Figure 12 (a) is essentially the same as that of Figure 3 (silk I structure). The primary structure of *A. pernyi* silk fibroin is very close to that of *S. c. ricini* silk fibroin [41,98]. Namely, the Ala residues present in polyalanine sequences or as the isolated ones in *A. pernyi* silk fibroin like *S. c. ricini* silk fibroin and the polyalanine sequences take α-helical conformation, whereas the isolated Ala residues take random coil in the liquid silk [99]. Thus, the assignment of the amide I band 1660 cm^−1^ of *A. pernyi* silk fibroin membrane as cast to α-helix in the IR spectrum is valid. On the other hand, the β-turn peak was assigned to 1690 to 1700 cm^−1^ in their paper because a small peak from1690 to 1700 cm^−1^ was assigned to β-turn conformation of the hairpin-folded antiparallel β-sheet structure [36]. However, the assignment of this band is not conclusively β-turn structure as is mentioned in the review of Vass et al. [38]. The amide I was 1655 cm^−1^ for *B. mori* liquid silk with silk I structure and was very close to the amide I band 1660 cm^−1^ for α-helix of *Antheraea pernyi* (*A. pernyi*) liquid silk, as shown in Figure 13. Therefore, many researchers concluded that silk I structure is α-helix or partially α-helix [23,24,25,26,27,28,29,30,31,32,33,34,35]. However, as described above, the silk I is type II β-turn structure, and the amide I 1655 cm^−1^ should be assigned to type II β-turn in the structural analysis of silk fibroin using the IR method. There are no sequences to be able to form α-helix in the primary structure of *B. mori* silk fibroin, as shown in Figure 1.

So far, many papers, including this one, assigned the silk I structures using IR and Raman spectroscopies by carefully considering type II β-turn model [52,53,100,101,102,103,104,105,106,107,108]. For example, Monti et al. [52,104] assigned the strong amide I, II, II, and V bands fell at 1654, 1540, 1240, and 660 cm^−1^ to type II β-turn structure in the IR spectrum of (AG)_15_ with silk I form. The IR method is very convenient for the structural analysis of silk fibroin and should use it more frequently qualitatively rather than quantitatively [37]. However, in these IR analyses, the experimental assignment of the amide I band to the β-turn structure has been done carefully by comparing the structure of peptides and proteins determined by another method, such as X-ray diffraction with the IR spectrum [38]. There are many papers to speculate silk I structure to be α−helix which are cited from previous papers repeatedly [23,24,25,26,27,28,29,30,31,32,33,34,35,36,37]. Although it is important to discuss the structure-property relationship of silk fibroin, the exact assignment of the IR band is clearly necessary to avoid serious mistakes. The silk I sample of (AG)_n_ took 100% type II β-turn structure, and therefore, all of the IR bands observed in Figure 3 are related to the type II β-turn structure. 

## 7. Conclusions and Future Aspects

In this review, the author showed that *Bombyx mori* silk fibroin structure before spinning (silk I) is type II β-turn, not α-helix or partially α-helix structure using several many solid-state NMR and solution NMR methods together with selectively stable isotope-labeled model peptides. On the other hand, many researchers have been reported that the structure of silk I is α-helix or partially α-helix using IR methods. The author emphasized that the conformational analyses of proteins and peptides from IR studies are not straightforward and should be very careful when the proteins contain β-turn structure, as pointed out by Vass et al. [38] using many experimental data. For example, Raman optical activity (ROA) and vibrational circular dichroism (VCD) can discriminate α-helix from other structures. Moreover, in the future, theoretical calculation of the IR bands for (AG)_n_ with type II β-turn structure will give a clear conclusion about the IR band assignment, especially for amide I.

## Figures and Tables

**Table 1:** Observed (I_obs_) and calculated (I_cald_) structure amplitudes for type II β-turn as the model of silk I [22,48,74]. Reprinted with permission from ref. 22. Modified from ref. 22.

Data No.	(*hkl*)	I*obs*	I*cald*	Data No.	(*hkl*)	I*obs*	I*cald*	Data No.	(*hkl*)	I*obs*	I*cald*
1	(011)	75	89	11	(122)	76	75	17	(043)	121	100
	(020)								(201)		
				12	(013)	49	20		(211)		
2	(021)	42	45		(140)				(004)		
									(220)		
3	(002)	151	164	13	(042)	115	108		(133)		
	(110)				(023)				(014)		
					(051)						
4	(012)	93	81		(141)			18	(221)	68	83
	(031)				(132)				(152)		
	(101)								(160)		
				14	(033)	58	46				
5	(111)	32	79		(103)			19	(230)	70	47
	(120)				(113)				(062)		
									(024)		
6	(022)	37	42	15	(150)	142	144				
					(052)			20	(161)	106	116
7	(121)	156	128		(142)				(231)		
	(040)				(060)				(202)		
					(151)				(212)		
8	(130)	42	35		(123)				(053)		
	(041)				(200)				(043)		
											
9	(032)	43	49	16	(210)	63	36	21	(034)	74	63
	(102)				(061)				(104)		
									(071)		
10	(112)	123	129								
	(131)										

**Table 2:** The intra- and intermolecular ^1^H-^1^H distances (in Å) of (AG)_15_ molecules with type II β-turn structure calculated using the coordinates of ^1^H atoms obtained from the CASTEP calculation.

	DQ Correlation		Intra-Molecular		Inter-Molecular	
①	G-Hɴ	A-Hɴ	2.83	(5 3)	3.43	(4 3)
②	G-Hɴ	A-Hα	2.12	(5 2)	3.46	(4 3)
③	G-Hɴ	G-Hα1	2.30	(5 3)		
④	G-Hɴ	G-Hα2	2.88	(5 3)	2.76, 3.42	(2 5),(4 3)
⑤	A-Hɴ	A-Hα	2.86, 3.52	(5 3),(5 2)	2.96	(4 3)
⑥	A-Hɴ	G-Hα1	3.28, 3.99	(5 3),(5 2)		
⑦	A-Hɴ	G-Hα2	3.47	(5 3)		
⑧	G-Hɴ	A-Hβ	3.89	(5 2)		
⑨	A-Hɴ	A-Hβ	2.87	(5 3)		
⑩	A-Hα	G-Hα1			3.23	(6 3)
⑪	A-Hα	G-Hα2			3.66	(9 4)
⑫	A-Hα	A-Hβ	2.70	(5 3)		
⑬	G-Hα1	A-Hβ	3.65	(5 4)	3.56	(2 4)
⑭	G-Hα2	A-Hβ			3.60	(3 4)
⑮	G-Hα1	G-Hα2	1.77	(5 3)		

The number of the DQ correlations ①–⑮ is the same as those in Figure 10. Reprinted with permission from ref. 86. Modified from ref. 86.
